# Young Hearts under Attack: The Alarming Increase in Heart Problems among Indian Youth

**DOI:** 10.2174/011573403X333367240925094017

**Published:** 2024-09-26

**Authors:** Priyanka Paul, Raj Kamal, Ankit Awasthi

**Affiliations:** 1 Department of Pharmaceutical Analysis, ISF College of Pharmacy, Moga, 142001, Punjab, India;; 2 School of Pharmacy, Desh Bhagat University, Mandi Gobindgarh, Punjab 147301 , India;; 3 Department of Pharmaceutics, Chitkara College Pharmacy, Rajpura, India

## 1 EDITORIAL

Heart disease, particularly heart attacks, is a growing health concern globally and in India. A heart attack, also known as a myocardial infarction, occurs when the blood flow to a part of the heart muscle is blocked, usually by a blood clot. This blockage can lead to damage or death of the heart muscle cells [1]. The most frequent and high-priority causes of heart attacks in young patients include premature atherosclerosis, familial hypercholesterolemia, substance abuse (especially cocaine and amphetamines), congenital or hereditary conditions (such as hypertrophic cardiomyopathy and coronary artery anomalies), inflammatory conditions (like Kawasaki disease), blood clotting disorders (thrombophilia), obesity/metabolic syndrome, stress-induced cardiomyopathy (Takotsubo syndrome), and other factors, such as oral contraceptive use and physical inactivity. Furthermore, arrhythmias, particularly those associated with sudden cardiac death, often result from conditions, such as hypertrophic cardiomyopathy, congenital coronary artery anomalies, and long QT syndrome. These irregular heart rhythms can disrupt the heart’s pumping function, leading to life-threatening situations. Ventricular fibrillation, characterized by chaotic quivering of the heart muscle, is the most common arrhythmia linked to sudden cardiac death. In young individuals, these arrhythmias can be triggered during physical exertion, stress, or even spontaneously, posing a risk of sudden death if not promptly addressed.

India has seen a significant increase in deaths due to heart attacks over the past three years, with a 12.5% rise in cases in 2022 alone, leading to 32,457 deaths, according to the National Crime Records Bureau (NCRB). This rise is attributed to the lingering effects of the COVID-19 pandemic and lifestyle factors. The Indian Heart Association reports that half of all heart attacks in Indian men occur under 50, and a quarter occur under 40. In 2020, heart attacks claimed the lives of 19,238 individuals aged 30 to 60, and in 2021, 2,541 deaths were reported among those aged 18 to 30 [2]. In 2023, the state of Gujarat in India experienced a surge in heart attack emergencies. The Emergency Management and Research Institute (EMRI) reported a 55% increase in heart-related emergency calls compared to the previous year, with 66,397 calls from January to November. The highest number of calls were during October and November, coinciding with the Navratri festival. Tragically, heart attacks claimed 2,853 lives in 2023, with a significant number of victims being between the ages of 11 and 25. This also includes a case of a class 10 student who suffered a fatal heart attack during an exam [3]. Further, 17-year-old girl, Sanjana Yadav, tragically suffered a fatal heart attack at her home in Indore despite having no family history of heart problems and having recovered from typhoid four months prior. The incident highlights potential risk factors, such as hypertension, high cholesterol, abnormal coronary arteries, and environmental triggers like cold weather or heart muscle weakness [4]. In another case, a 23-year-old man named Jathin from Haryana tragically passed away due to a heart attack during a trek to Tadiandamol, the highest peak in Kodagu, near Napoklu in Madikeri taluk. He was part of a six-member team that had arrived from Bengaluru for the trek. Upon reaching the peak, Jathin experienced breathing difficulties and collapsed. Despite attempts by a doctor from another trekking team to revive him, Jathin succumbed to a severe heart attack [5].

A 29-year-old academic was found unconscious and later tested positive for COVID-19. Despite initial treatment, his condition worsened, and he was declared deceased upon arrival at the emergency room. A post-mortem CT scan was performed due to suspicions from family members and a lack of standard autopsy infrastructure. The scan revealed an intracerebral bleed, likely due to a severe hypertensive episode, and cerebral edema with bilateral tonsillar herniation, which were determined to be the cause of death. This case demonstrated the potential of post-mortem CT as a viable alternative to traditional autopsies, particularly in reducing the risk of exposure to infectious agents [6].

A 34-year-old man unexpectedly passed away due to a widespread aortic dissection, a condition typically seen in older individuals. This case was unique as it involved an extensive dissection from the root to the descending aorta, including a retrograde extension to the brachiocephalic and left common carotid arteries. The patient initially presented with breathlessness and chest pain, and despite medical intervention, he suffered a cardiac arrest. This case underscores the need for considering aortic dissection as a potential diagnosis in young adults presenting with severe pain and the critical importance of prompt medical response in such situations [7].

A 22-year-old Saudi female presented to the emergency department with sudden onset chest pain radiating to both arms and associated with palpitations, nausea, and shortness of breath. Past history revealed similar symptoms six months prior, but no medical attention was sought. Despite interventions, including cardiac catheterization and multiple resuscitation efforts, the patient succumbed to recurrent cardiac arrest. With no traditional risk factors and inconclusive investigations due to her short stay, her unfortunate death underscores the complexity of sudden cardiac events in young individuals without apparent risk factors [8].

Two young adults, aged 25 and 28, had experienced out-of-hospital cardiac arrest (OHCA) following energy drink (ED) consumption. Conventional resuscitation was initiated, followed by transfer to the center for immediate extracorporeal cardiopulmonary resuscitation (eCPR) using a Lund University Cardiopulmonary Assist System (LUCAS). Studies have consistently linked ED consumption to elevated blood pressure, platelet aggregation, and arrhythmias, with documented cases of ventricular fibrillation and myocardial infarction post-ED intake. While underlying mechanisms remained unclear, caffeine and other ingredients were likely to have affected the sympathetic and cardiovascular systems. Heightened awareness among healthcare providers and intensified regulation of ED consumption have been warranted to mitigate associated cardiovascular risks [9].

The increase in heart problems among young adults is linked to unhealthy lifestyles, stress, smoking, drinking, and poor diet. The pandemic has also contributed to a rise in cardiovascular issues. Young adults may experience atypical symptoms, making early detection difficult. The broader impact of heart attacks on younger generations extends to economic burdens on healthcare systems and public health. Heart attack management involves a combination of medications, procedures, and therapy [10]. Medications like aspirin, nitroglycerine, thrombolytics, and supportive medicines are used to reduce clotting, maintain blood flow, and reduce strain on the heart. Procedures, such as coronary angioplasty and stenting, as well as coronary artery bypass graft (CABG), are performed to restore blood flow to the heart. Additionally, cardiac rehabilitation programs involving exercise, lifestyle changes, and diet are recommended. These treatments are administered under the supervision of a healthcare professional, and immediate medical attention is advised if heart attack symptoms are observed [11].

## FUTURE PERSPECTIVE AND CONCLUSION

Addressing this issue requires a multifaceted approach. Health education, promoting healthy lifestyles, and improving access to preventive healthcare are key. These include quitting smoking, which can reduce the risk of heart disease and stroke almost immediately. Regular exercise, aiming for 150 minutes of moderate or 75 minutes of vigorous activity per week, is also crucial. A balanced diet low in saturated and trans fats and sodium and high in fruits, vegetables, whole grains, nuts, and fish is recommended. Maintaining a healthy weight can help regulate blood pressure, cholesterol, triglycerides, and blood sugar levels. Adequate sleep, at least seven hours per night, and stress management techniques, such as physical activity, relaxation exercises, mindfulness, yoga, and meditation, can also contribute to heart health. Regular health screenings for risk factors, such as blood pressure, cholesterol, blood sugar, heart disease, and diabetes, are important. Lastly, preventing infections through daily dental hygiene, vaccinations against COVID-19 and other diseases, and seeking treatment for any illnesses that may affect the heart is advised.

## LIST OF ABBREVIATIONS

CABG: Coronary Artery Bypass Graft
eCPR: Extracorporeal Cardiopulmonary Resuscitation
ED: Energy Drink
EMRI: Emergency Management and Research Institute
LUCAS: Lund University Cardiopulmonary Assist System
NCRB: National Crime Records Bureau
OHCA: Out-of-Hospital Cardiac Arrest
